# Immunoprofiling: An Encouraging Method for Predictive Factors Examination in Lung Cancer Patients Treated with Immunotherapy

**DOI:** 10.3390/ijms22179133

**Published:** 2021-08-24

**Authors:** Kamila Wojas-Krawczyk, Iwona Paśnik, Tomasz Kucharczyk, Irena Wieleba, Natalia Krzyżanowska, Michał Gil, Paweł Krawczyk, Janusz Milanowski

**Affiliations:** 1 Department of Pneumonology, Oncology and Allergology, Medical University of Lublin, 20-605 Lublin, Poland; tomasz.kucharczyk@umlub.pl (T.K.); irena.wieleba@umlub.pl (I.W.); natalia.krzyzanowska@umlub.pl (N.K.); janusz.milanowski@umlub.pl (J.M.); pawel.krawczyk@umlub.pl (P.K.); 2 Department of Clinical Pathomorphology, Medical University of Lublin, 20-605 Lublin, Poland; iwona.pasnik@umlub.pl; 3 Institute of Genetics and Immunology GENIM LCC in Lublin, 20-609 Lublin, Poland; genim@tlen.pl

**Keywords:** immunophenotyping, tumor microenvironment, immune checkpoints, immunotherapy

## Abstract

The efficiency of immunotherapy using monoclonal antibodies that inhibit immune checkpoints has been proven in many clinical studies and well documented by numerous registration approaches. To date, PD-L1 expression on tumor and immune cells, tumor mutation burden (TMB), and microsatellite instability (MSI) are the only validated predictive factors used for the qualification of cancer patients for immunotherapy. However, they are not the ideal predictive factors. No response to immunotherapy could be observed in patients with high PD-L1 expression, TMB, or MSI. On the other hand, the effectiveness of this treatment method also may occur in patients without PD-L1 expression or with low TMB and with microsatellite stability. When considering the best predictive factor, we should remember that the effectiveness of immunotherapy relies on an overly complex process depending on many factors. To specifically stimulate lymphocytes, not only should their activity in the tumor microenvironment be unlocked, but above all, they should recognize tumor antigens. The proper functioning of the anticancer immune system requires the proper interaction of many elements of the specific and non-specific responses. For these reasons, a multi-parameter analysis of the immune system at its different activity levels is considered a very future-oriented predictive marker. Such complex immunological analysis is performed using modern molecular biology techniques. Based on the gene expression studies, we can determine the content of individual immune cells within the tumor, its stroma, and beyond. This includes all cell types from active memory cytotoxic T cells, M1 macrophages, to exhausted T cells, regulatory T cells, and M2 macrophages. In this article, we summarize the possibilities of using an immune system analysis to predict immunotherapy efficacy in cancer patients. Moreover, we present the advantages and disadvantages of immunoprofiling as well as a proposed future direction for this new method of immune system analysis in cancer patients who receive immunotherapy.

## 1. Introduction

An effective immune response is a particularly complex interaction between the cellular elements of the immune system and the cytokines released by them [1]. We must remember that these proper interactions are extremely important, lying at the heart of our anticancer defenses. In short, the successful anti-tumor response involves two branches of the immune system: non-specific cells, which are intended to recognize the tumor cell antigen, phagocytose it, and present it to cytotoxic T lymphocytes (CTLs) in peripheral lymph nodes [1,2,3]. From that point, the specific anti-tumor response begins to develop, which should be guaranteed by properly mobilized T cells. Activated T cells should infiltrate the neoplastic tissue and recognize the neoplastic antigens. At the site of its recognition, the intracellular cytotoxic proteins are released from CTLs and, together with non-specific mechanisms provided by macrophages or NK cells, the cancer cells can2 be ultimately eliminated. The intercommunications between the cancer microenvironment and the immune system constitute an undoubtedly heterogeneous and dynamic process, influencing the course of the disease [1,4,5].

Nowadays, it is very well established that immunotherapy with immunological checkpoint inhibitors (ICIs) has revolutionized cancer treatment, especially for patients without actionable driver mutations [6,7]. Three groups of ICIs are widely used in different cancer treatments. The first group consists of anti-PD-1 (Programmed Death 1) antibodies, which include pembrolizumab and nivolumab, and block the programmed death 1 receptor on the lymphocyte surface, resulting in the increased activity of these cells. The second group of ICIs is anti-PD-L1 antibodies, including atezolizumab, durvalumab, and avelumab, which block the ligand for PD-1-PD-L1-on tumor cells and on tumor-infiltrating immune cells [6,7,8]. The third group is anti-CTLA-4 (Cytotoxic T Lymphocyte Antigen 4) antibodies, mainly ipilimumab, which blocks the interaction between CTLA-4 on lymphocytes and B7-1 or B7-2 on antigen-presenting cells (APCs), restoring the main lymphocyte stimulating signal from CD28 [6,7,8].

In many clinical trials, it has been clearly indicated that PD-L1 protein expression is an important factor for stratifying patients to receive ICIs and to reach clinical efficacy [9,10]. However, it should also be noted that the expression of the PD-L1 molecule is not an ideal marker, and among the many disadvantages associated with its technical staining, the most important seems to be a heterogeneous expression of this molecule through the tumor, its variability between primary and metastatic sites, and its dependence on the history of treatment (chemotherapy and radiotherapy could change PD-L1 expression on tumor cells) [11,12,13,14]. We should also mention at this point the tumor mutational burden (TMB), the predictive factor associated with much hope as a potentially important biomarker in qualification for ICIs therapy [15,16]. The high number of somatic mutations detected in cancer tissue causes an increased number of neoantigens, which translates into increased immunogenicity of tumors. It would seem that TMB could serve as a link between both genetic information regarding the presence of abnormal genes in cancer tissue and the activation of the immune system caused by these abnormalities [15,16,17]. However, ultimately, this factor did fulfill all aspirations associated with it. Indeed, a CheckMate-568 study, where the effectiveness of combination therapy (nivolumab and ipilimumab) in untreated advanced NSCLC patients was examined, demonstrated that there was no evidence of increased immunotherapy efficacy in patients with very high TMB (≥15 mut per Mb) compared to patients with high TMB (≥10 mut per Mb) [18].

An important group of predictors could be the markers determined in peripheral blood, which is a relatively available material [19]. The most investigated serum soluble biomarker is blood tumor mutational burden (bTMB), estimated by commercial platforms (e.g., the FoundationOne CDx assay) in cell-free DNA (not in peripheral blood circulating cancer cells) [19]. The conventional signs of inflammation tested in peripheral blood, such as LDH, C-reactive protein (CRP), or IL-6 concentration, could be considered as reliable biomarkers of ICIs effectiveness. However, the current data indicate only retrospective analysis of inflammatory-associated factors in cancer-bearing patients who received immunotherapy. Moreover, the neutrophil-to-lymphocyte ratio (NLR), which could be calculated easily from a complete blood testing report, or the systemic immune-inflammation index (SII), which combines NLR and platelet-to-lymphocyte ratio (PLR), may be the markers of early progression in ICIs therapy. It was demonstrated that NLR could distinguish between non-responders and responders to nivolumab therapy at an early stage of treatment [20,21,22].

Undoubtedly, after proper qualification of patients for immunotherapy based on registration rules, the proper activity of the host immune system is of great importance to obtain clinical benefits from this therapy [22]. To specifically stimulate lymphocytes, not only should their activity in the tumor microenvironment be unlocked, but above all, they should recognize tumor antigens. The proper functioning of the anticancer immune system requires the appropriate interaction of many elements of the specific and non-specific responses. Consequently, the pre-existing immunity in the tumor site determines the survival of immunized patients and the chances of responding to immunotherapy [23,24,25]. Therefore, we should consider whether immunological profile analysis in cancer tissue may be a reliable biomarker in the prospective qualification for immunotherapy.

## 2. Quantity or Quality of Tumor Infiltration as an Independent Prognostic Factor in Cancer Patients

The infiltration of immune system cells into neoplastic tissue has been investigated for many decades [22,23,24,25]. The first studies to assess the state of the immune system were conducted in melanoma, colorectal cancer, head and neck cancer, or breast cancer [26,27]. These studies documented a positive correlation between the density of immune system cell infiltration into neoplastic tissue and the prognosis of cancer patients. Usually, the intensity of neoplastic tissue infiltration positively correlated with the clinical prognosis [28,29,30]. The best example of this phenomenon was observed for patients with melanoma. High intratumoral numbers of CD3-positive, CD4-positive, and CD8-positive lymphocytes as well as high expression of the receptor for interleukin 2 (CD25) on these cells were related to a favorable outcome for melanoma patients [23,31]. However, for renal cell and prostate carcinoma, strong T cell infiltrates were associated with worse outcome, which was probably caused by the absence of tumor-specific T cells in the tumor microenvironment [32,33]. It is also possible that these cells are functionally suppressed in the tumor microenvironment (e.g., mutations in *JAK1* and *JAK2* genes in tumor cells can lead to inappropriate antigen presentation). In addition, these lymphocytes could express other negative immune checkpoints, e.g., LAG-3 (Lymphocyte Activation Gene-3) or TIM3 (T Cell Immunoglobulin 3) on their surface or be extinguished by the interaction with myeloid-derived suppressor cells [32,33,34].

A similar situation was observed for lung cancer patients. In many studies, the presence of tumor infiltrated lymphocytes (TIL) has been defined as a favorable prognostic factor [35,36,37,38]. However, a detailed analysis of the immunophenotype of lymphocytes infiltrating the lung adenocarcinoma tissue in stage I showed that a high density of FoxP3 (Forkhead Box P3)-positive lymphocytes and high stromal FoxP3^+^ cells/CD3^+^ cells ratio was a strong predictor of recurrence [38]. Moreover, a high expression of IL-7R on tumor cells was considered as a significant (*p* = 0.001) marker for poor overall survival, while a high expression of tumor interleukin 12 receptor β2 was associated with a better outcome (five-year recurrence-free probability; *p* = 0.026) for patients with stages IA and IB [38]. The authors suggested that, based on the biology of the tumor immune microenvironment, patients could be stratified for immunotherapeutic intervention [38]. In advanced adenocarcinoma patients who received chemotherapy, Kawai et al. performed immunohistochemical analysis of CD68^+^ macrophages, c-Kit^+^ mast cells, and CD8^+^ lymphocyte infiltration in cancer stroma and nests [39]. Patients with more tumor-infiltrating macrophages as well as lymphocytes detected in cancer nests showed significantly better survival than patients with cancer stroma predominant infiltration. However, no significant correlation was found between the number of immune cells in either cancer nests or stroma and chemotherapy response. On the contrary, very interesting research was presented by Kinoshita et al. showing that CD8-positive T cells in the tumor microenvironment of non-smoking lung cancer patients were defined as a poor prognostic factor. These cells were less activated, had an immunodysfunctional phenotype and expressed the high level of various immunoregulation genes. In contrary, high infiltration of activated CD8-positive T cells expressing interferon gamma and granzyme B was correlated with postoperative survival in those patients [36].

**Figure 1:**
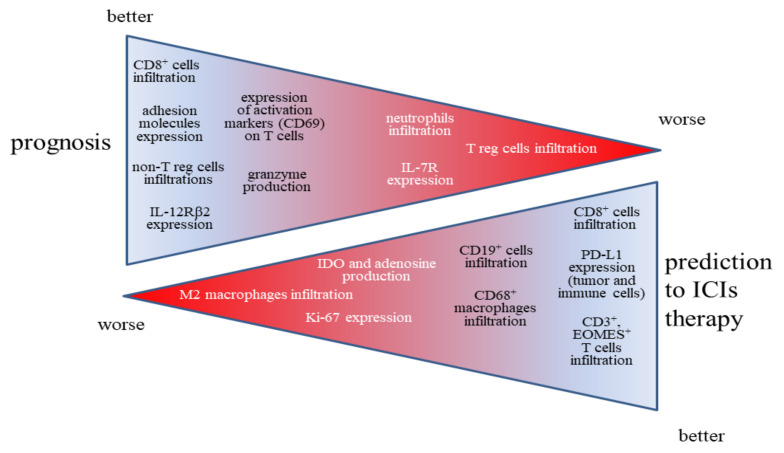
The summary of the immune markers associated with the prognosis and with the prediction of response to immunotherapy of lung cancer patients.

It was clearly shown that the information regarding immune system status in tumor tissue may be of fundamental importance in predicting the prognosis of lung cancer patients [40,41,42]. However, all these considerations obviously indicate that the information about the absolute number of T cells is not enough to assess the patient’s prognosis, but the information about the functioning of T lymphocytes and the presence of other immune system cells in the cancer tissue is necessary. A summary of the immune markers associated with the prognosis of lung cancer patients along with the prediction of response to immunotherapy are presented in Figure 1. To summarize, it is not only the presence of the immune system in tumor tissues (quantity), but also the kind of immune cell infiltration (quality) that is of great importance for the cancer prognosis and the clinical benefits from immunotherapy.

## 3. Three Different Immunoprofiles of Tumor Tissue

It has been indicated, using data regarding ICIs effectiveness in various malignancies, that tumors have three distinct immunoprofiles based on their immune system activation: (1) “hot” tumors, which are strongly infiltrated with T lymphocytes and with activation of different inflammatory signals; (2) “cold” tumors, which are scanted of any immune cells infiltration nor inflammatory signs; (3) tumor with immune exclusion, where immune cells are at the periphery or within the stromal tissue [43,44,45].

The “hot” tumors, also described as highly inflammatory tumors, are thus defined by the presence of strong inflammation signals within the tissue, both in the form of inflammatory cell infiltration and high levels of pro-inflammatory cytokines (Figure 1). The tumor cells in “hot” tissue have undergone many mutations that create neoantigens, which should be recognized by the immune cells [45,46]. However, despite the fact that the neoplastic tissues are very intensively infiltrated by the immune system cells of specific and non-specific responses, the immune response in this type of tumor is extremely ineffective.

**Figure 2:**
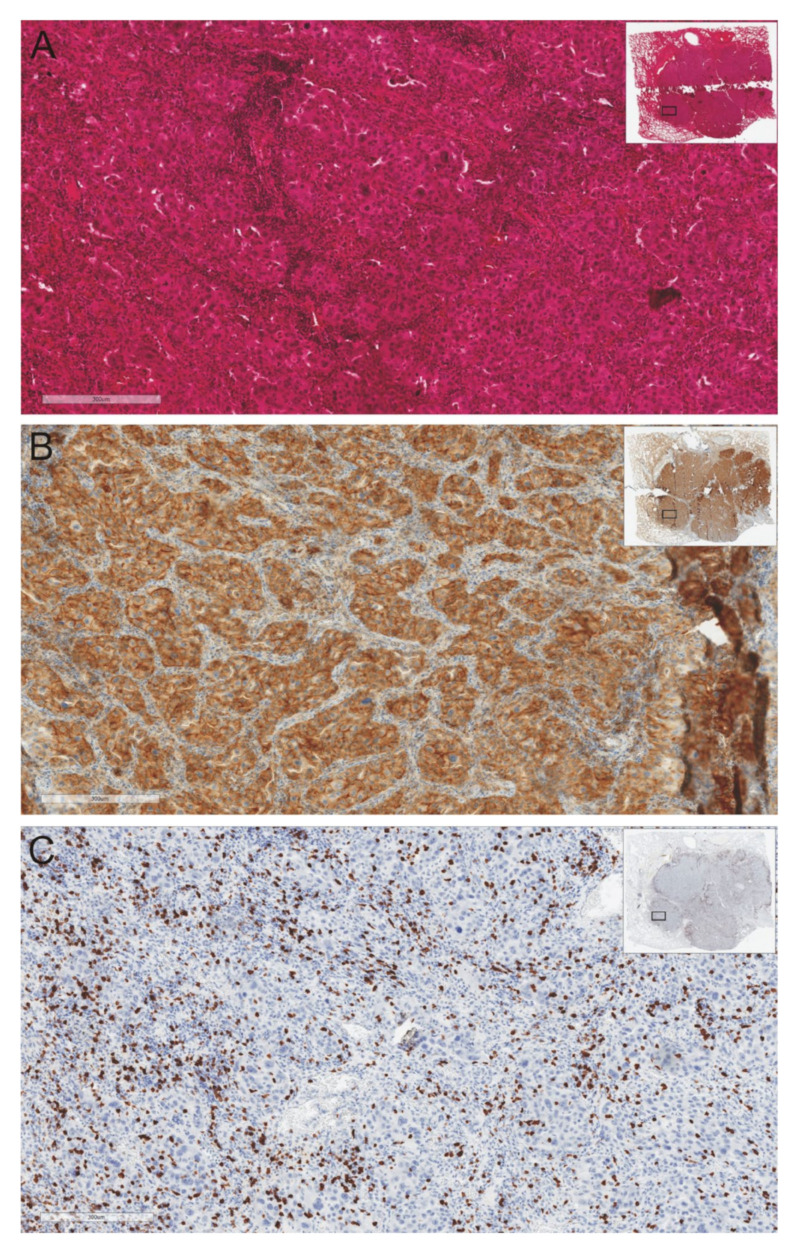
A case illustration of lung adenocarcinoma tissue with strong infiltration of CD8-positive lymphocytes and high expression of PD-L1 on tumor cells: (**A**). hematoxylin/eosine staining; (**B**). PD-L1 expression; (**C**). CD8 expression. Immunohistochemical staining was performed on authors’ own materials.

Firstly, the high percentages of cytotoxic T lymphocytes are widely described. However, they are functionally inactive [44,45,46]. In a mouse tumor model, at least four subpopulations of tumor-infiltrating T cytotoxic lymphocytes were labeled: (a) T lymphocytes with a functionally depleted cell phenotype with a very high expression of negative immune checkpoint molecules such as PD-1, LAG-3, TIM3; (b) terminally differentiated T lymphocytes with an activated phenotype with intermediate expression of PD-1, LAG-3, and TIM3; (c) T lymphocytes at an early stage of differentiation with a low expression of PD-1 molecule, an intermediate expression of intracellular transcription factor T-bet which affects their differentiation into T-helper type 1 (Th1) cells, and with an intermediate expression of proteins inhibiting apoptosis (e.g., Bcl-2); (d) apoptosis-resistant migratory T lymphocytes with high expression of intracellular Bcl-2 protein, with high expression of cell adhesion molecule (L-selectin, CD62L), and with a lack of any inhibitory checkpoint molecules [47,48]. Moreover, regulatory T lymphocytes (Treg) with a high expression of the intracellular transcription factor FoxP3 and with a strong ability to secrete TGF-β (transforming growth factor beta) are also observed [47,48,49]. In relation to the cells involved in the development of the non-specific immune response, “hot” tumors are also strongly infiltrated by tumor-associated macrophages (TAMs), especially type 2 macrophages, which exhibit pro-tumor attributes [50,51]. M2 macrophages secrete IL-10, TGF-β, and other anti-inflammatory cytokines, which have the immunosuppressive function of reducing inflammation and contributing to tumor growth. The ability of TAMs to lyse tumor cells and to present tumor-associated antigens is decreased, causing a reduction of stimulation of the anti-tumor functions of T and NK cells [50,51,52]. In particular, lymphocytes lose their ability to produce interferon-gamma (INF-γ)–a cytokine with an important significance in the tumor microenvironment influencing the PD-L1 expression on tumor cells [53,54]. Patients with “hot” tumors are ideal candidates for ICI therapy due to the presence of T cell infiltrates at the tumor tissue and the usually high expression of PD-1 on lymphocytes and PD-L1 on tumor or immune cells (Figure 2).

**Figure 3:**
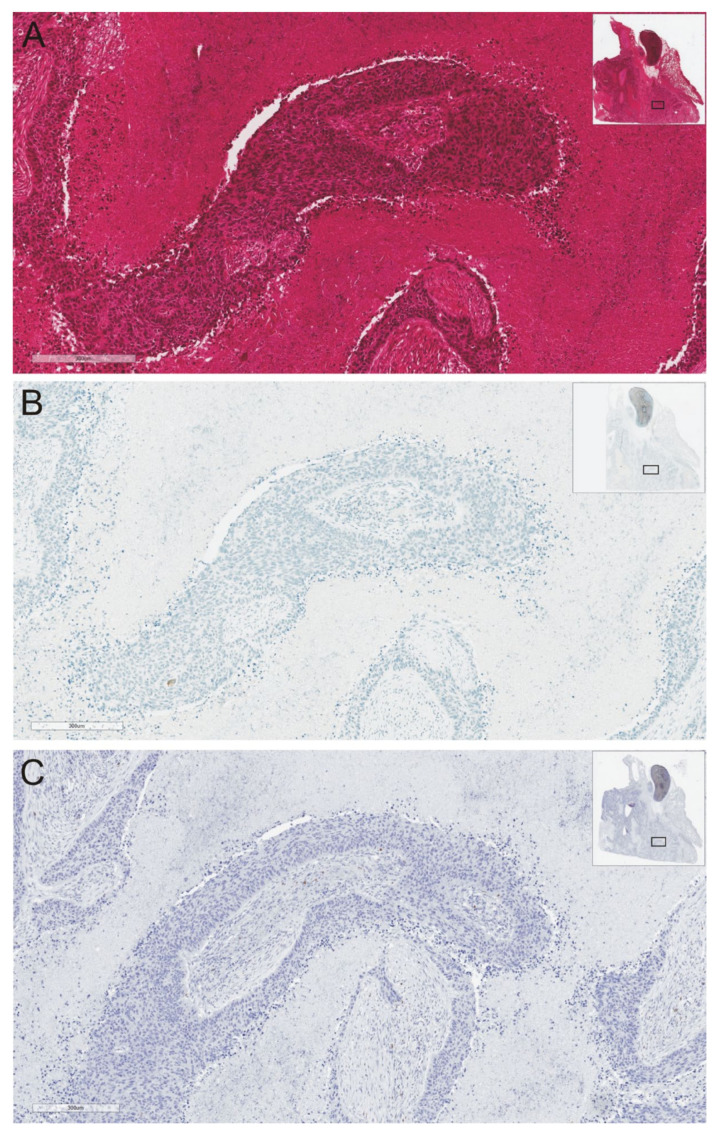
A case illustration of lung adenocarcinoma tissue without CD8+ lymphocyte infiltration and lack of PD-L1 expression on the tumor cells: (**A**). hematoxylin/eosine staining; (**B**). PD-L1 expression; (**C**). CD8 expression. Immunohistochemical staining was performed on authors’ own materials.

The “cold” tumors, also described as non-inflamed tumors, are thus defined by the absence of any inflammation signals within the tissue [43]. This type of cancer is not infiltrated by any immune system cellular elements, furthermore, high concentrations of pro-inflammatory cytokines and products of tumor tissue metabolites, e.g., NO (nitric oxide), IDO (Indoleamine 2,3-Dioxygenase), and arginase are not observed in the microenvironment. Moreover, neither high mutational burden nor the presence of neoantigens are observed in a “cold” tumor, and it is postulated that cold tumors are more likely to bear a single driver mutation [43]. Patients with the driver mutation and a “cold” tumor respond better to molecularly targeted therapies than to immunotherapy. Some authors describe that the microenvironment surrounding “cold” tumors could contain some myeloid-derived suppressor cells (MDSC) and Treg cells, which are known to dampen the immune response and inhibit T cells trying to move into the tumor [52,55]. MDSC and regulatory T cells inhibit the maturation of dendritic cells in the tumor and, together with a low amount of adhesion molecules (CD34, E-selectin, vascular cell adhesion molecule (VCAM), and intercellular adhesion molecule (ICAM)), stimulate blood vessel formation and reduce cell adhesion as well as the promotion of metastasis [43,52,55]. However, the presence of even a small infiltration of neoplastic tissue by the immune system cells prompts us to define this type of tissue as a tumor with immune exclusion (Figure 3).

**Figure 4:**
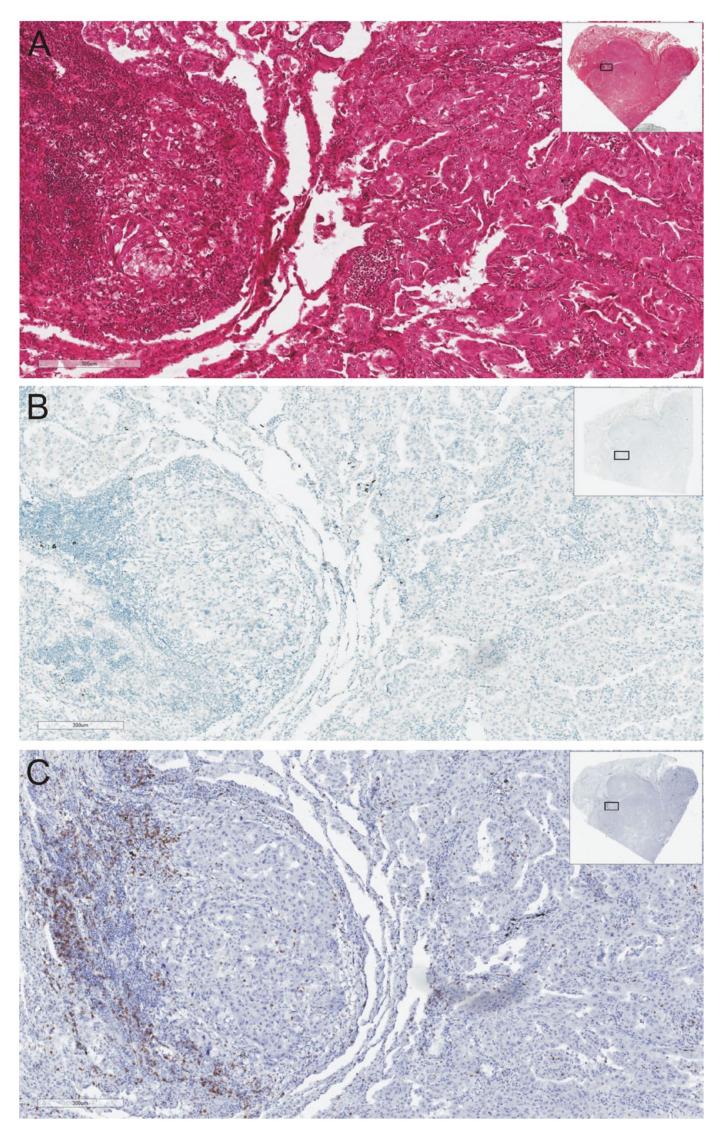
A case illustration of lung adenocarcinoma tissue in which the cells of the immune system marginally infiltrate the neoplastic tissue without PD-L1 expression: (**A**). hematoxylin/eosine staining; (**B**). PD-L1 expression; (**C**). CD8 expression. Immunohistochemical staining was performed on authors’ own materials.

Tumors with immune exclusion are also defined as tissues where immune cells exhibit the deficit of homing to the tumor bed [56]. Immune-excluded tumors refer to tumors with a strong immune suppressed tumor microenvironment (TME) represented by T cells clearly embedded in the tumor periphery with high TGF-beta signaling, myeloid inflammation, and angiogenesis [56,57]. Pai et al. proposed different mechanisms probably responsible for immune exclusion. Firstly, there are mechanical barriers which prevent direct contact between T cells and cancer cells [56]. The predominant role appears to be played by excessive activity of VEGF produced by the tumor cells and signaling pathways activated by VEGF [56,57,58]. Secondly, functional barriers that consist of biological and metabolic interactions between cancer cells, stromal, and immune cells may exist [56]. Mutations in the genes which encode molecules in the signaling pathways of tumor cells are probably responsible for a very strong suppression of the immune cells’ influx into the neoplastic tissue [59,60]. Molecular abnormalities include incorrect activation of Wnt/β-catenin signaling pathways, which inhibits the tumor tissue infiltration with CD103-positive dendritic cells as well as causing loss of PTEN pathways’ activating elements, which further inhibits cytotoxic T cells invasion [56,59,60,61]. In this type of tumor, cancer cell antigens have been recognized, the specific immune response has been induced, but the extremely strong immunosuppressive tumor microenvironment (high concentration of tumor tissue metabolites: NO, IDO, and arginase) does not allow the immune cells to penetrate the neoplasm (Figure 4). ICI therapy is usually ineffective in patients with tumors with immune system exclusion. Therefore, there are reasons to combine ICIs with other methods of immunotherapy in patients with this type of tumor.

Based on immune system status analysis in the three types of tumors described above, it seems obvious that each of them requires a distinct therapeutic approach. Unfortunately, in lung cancer patients, the analysis of the immune system in cancerous tissue has never been used as a predictive factor for qualification to ICI therapy in prospective clinical trials. These studies were performed only in retrospective analysis.

## 4. Immunoscoring of Lung Tumor Tissue as a Predictive Factor for Qualification to ICIs Therapy

The better efficacy of immunotherapy in patients with “inflamed” tumors than in patients with “non-inflamed” tumors seems to be well documented in the literature [40,44,62,63]. The results obtained in melanoma patients treated with immunological checkpoint inhibitors indicated that tumor regression after PD-1 blockade requires tumor infiltration by CD8-positive cells. Higher numbers of CD8-positive and PD-1-positive cells at the invasive tumor margin and inside the tumor were found in pre-treatment tumor samples in those patients who had responded to pembrolizumab therapy [62,63].

In prospective trials regarding lung cancer patients treated with immunotherapy, immunophenotyping of tumor tissue has never been used as an ICI predictor. One of the few retrospective trials where the activity of the immune system was assessed in tumor tissue was the POPLAR study [64,65]. In this clinical trial, the efficacy of atezolizumab was compared with docetaxel in the second-line treatment of locally advanced or metastatic NSCLC patients. The immune gene signature profiles, mainly those associated with immune cell activation (e.g., INF-γ signaling, immune cytolytic activity) were tested by next-generation sequencing (NGS) in tumor tissue and the results were correlated with the immunotherapy outcome. The higher expression of genes associated with T-effector activation (*CD8A*, *GZMA*, *GZMB*, *IFN-γ*, *EOMES*, *CXCL9*, *CXCL10*, and *TBX21*) was significantly associated with clinical benefits in patients receiving anti-PD-L1 immunotherapy, and the pre-existing primed immune response was observed in those tissue samples [64,65]. It seems that “hot” tumors, which are associated with denser PD-1-positive T lymphocyte infiltration and with high gene expression for pro-inflammatory factors, are more sensitive to anti-PD-1 or anti-PD-L1 blockade when used as monotherapy.

Modern genetic techniques, such as next-generation sequencing, are widely used for searching for driver mutations in many genes. Based on the results of these examinations, patients could be qualified into selected targeted therapy or into original clinical trials with innovative therapies yet unregistered [66]. However, it is extremely rare that next-generation sequencing is carried out to obtain information about the immune system status in the neoplastic tissue.

One of the most important trials regarding the correlation between immunotherapy efficacy and the immune landscape in lung cancer tissue was presented by Hwang et al. [66,67]. A panel of 395 immune-related genes was tested in pretreatment tumor samples using an Oncomine Immune Response Research Assay. Advanced non-small cell lung cancer patients receiving anti-PD-1 immunotherapy were classified into two groups according to their response to ICIs: patients with durable clinical benefit (DCB) and patients with non-durable clinical benefit (NDCB). Firstly, the proportion of core tumor-infiltrating lymphocytes was significantly higher in the DCB than in the NDCB. Surprisingly, the median TMB did not significantly differ between those two groups. The most important results are that the best impact on discrimination between DCB and NDCB patients had specific gene signatures defined as macrophage M1 signature and peripheral T cell signature. The group M1 consists of high expression of the following genes, namely *CCR7*, *CD27*, *CD48*, *FOXO1*, *HLA-B*, *HLA-G*, *LAMP3*, and *NFKBIA*, while the peripheral T cell signature included high expression of *HLA-DOA*, *GPR18*, and *STAT1* genes. Moreover, among so many tested genes, the authors found that the highest expression of *CD137* and *PSMB9* was characteristic for durable clinical benefit from anti-PD-1 immunotherapy. The short summary of the work by Hwang et al. showed very evidently that an effective immune response in neoplastic diseases is an overly complex interaction. Therefore, integrated multigene signature analysis seems to be a better predictor than single PD-L1 expression or TMB status assessment. Moreover, this study also proved that significant efforts should be made to obtain the proper information about the following two branches of the immune response: non-specific (M1 signature) and specific (peripheral T cell signature) activity of the immune response [67].

A new solution to determine the immunological predictive factors, such as PD-L1 expression and the presence of CD8-positive T cells, is automated image analysis, as performed by Althammer et al. Archival or fresh tumor biopsies were digitally scored for PD-L1- and CD8-positive cells densities across multiple tumor types, including NSCLC patients who received anti-PD-L1-based immunotherapy. Median overall survival for patients who were treated with durvalumab was 21 months for CD8- and PD-L1-double positive signature, while for CD8- and PD-L1-negative signature it was only 7.8 months (*p* = 0.00002). The authors concluded that CD8- and PD-L1-double positive signature provided greater stratification of OS than single high densities of CD8-positive cells or PD-L1-positive cells, but only for ICI-treated patients. However, for immunotherapy-naïve patients, a single high density of CD8-positive cells was significantly associated with higher median overall survival (67 months) than for the group with low CD8-positive cell density (39 months; *p* = 0.0009) [68].

Unfortunately, in the literature, there remain insufficient data indicating the usefulness of determining the basic immunological biomarkers in predicting the response to ICIs of lung cancer patients.

## 5. Methodology

Expression of PD-L1 (clone SP263, Ventana, Arizona, USA) and CD8 (clone SP239, Spring Bioscience, Pleasanton, California, USA) was evaluated using immunohistochemistry technique in BenchMark GX autostainer according to the manufacture’ instruction. The pathomorphological evaluation was carried out by two independent observers.

## 6. Conclusions

The anti-cancer immune response is an extremely complex process which requires the perfect interaction of cellular elements belonging to the two branches of the immune response: specific and non-specific. Immunotherapy with antibodies blocking negative immunological checkpoint molecules restores the activity of exhausted T cells. However, the clinical effectiveness of this method depends not only on the T lymphocyte function restoration, but also on the proper recognition of the tumor antigen, the amounts of immune cells in the tumor tissue, and the interaction between the tumor and the immune cells. In such a complex interaction, it seems that the determination of a single predictive biomarker may not be sufficient. Indeed, the presence of PD-L1 expression on tumor cells, one of the best tested predictive factors, does not guarantee immunotherapy success for NSCLC patients.

In the presented review, the authors attempted to show that the elementary information about the presence and activity of the immune system in neoplastic tissue could be defined as a strong predictive factor for patients undergoing immunotherapy. Simple immunological analysis of the existing immune response in the cancer tissue, for example, the presence of CD8-positive lymphocytes as well as CD68-positive macrophages, and their localization inside the tumor, could be added into the basic pathomorphological diagnosis of NSCLC patients. This is a relatively quick and inexpensive technique that could give the oncologists important information about a pre-existing immune response. However, more advanced techniques, such as NGS, performed to examine the expression of genes encoding pro- and anti-inflammatory factors, should also undergo a process of evaluation in order to be implemented in clinical practice for the selection of patients for immunotherapy.

The understanding of critical molecular mechanisms involved in the complexity of immune system-tumor communication allows us to provide an appropriate therapeutic approach and prognose the disease outcome. Generally, cytotoxic T cells, memory T cells, and Th1 cells are associated with prolonged survival, while high densities of regulatory T cells, myeloid-derived suppressor cells (MDSCs), or neutrophils are usually related with poor prognosis. Double positive PD-L1 and CD8 signature, as well as multiparameter immunological analysis of tumor tissue, has proven strong predictive value of response to immunotherapy. However, the future direction of immunological assays is not only focused on prognostic and predictive biomarkers analysis, but also “mechanistic immune signature” and “escape signature” markers are of importance [69]. A mechanistic immune signature consists of specific immune genes, predominantly associated with cytotoxic T cells activity, which expression is elevated significantly in immunotherapy-responding patients. Moreover, it was recently demonstrated that anti-PD-1 immunotherapy efficacy is dependent on intra-tumoral chemokines CXCL9–CXCR3 axis activity. Regarding to escape biomarkers, defects in major signaling pathways associated with PD-L1 expression are the most frequently described. In melanoma patients, loss-of-function mutations in IFN-gamma encoding genes or Janus kinase 1 (JAK1) and JAK2 are associated with acquired resistance to anti-PD-1 therapy. The analysis of these parameters in the tumor tissue at the start of treatment would certainly turn attention to the possibility of early progression in these patients. However, we should remember that there is no clear and obvious frontier between the described types of immunological biomarkers [69].

In conclusion, the knowledge about the immune system status within the neoplastic tissue is extremely important information. We can use simple immunohistochemical staining techniques to determine the presence and activity of immune system cells. The next step is to introduce and promote basic immunological parameter examination in the routine pathomorphological diagnostics, which will certainly translate into more effective patient qualification for immunotherapy and further monitoring of the treatment course.

## References

[1] Galon J., Bruni D. (2020). Tumor Immunology and Tumor Evolution: Intertwined Histories. Immunity.

[2] Chen D.S., Mellman I. (2017). Elements of cancer immunity and the cancer-immune set point. Nature.

[3] Chen D., Mellman I. (2013). Oncology meets immunology: The cancer-immunity cycle. Immunity.

[4] Dunn G.P., Bruce A.T., Ikeda H., Old L.J., Schreiber R.D. (2002). Cancer immunoediting: From immunosurveillance to tumor escape. Nat. Immunol..

[5] Schreiber R.D., Old L.J., Smyth M. (2011). Cancer immunoediting: Integrating immunity’s roles in cancer suppression and promotion. Science.

[6] Hargadon K.M., Johnson C.E., Williams C.J. (2018). Immune checkpoint blockade therapy for cancer: An overview of FDA-approved immune checkpoint inhibitors. Int. Immunopharmacol..

[7] Korman A.J., Peggs K.S., Allison J. (2006). Checkpoint blockade in cancer immunotherapy. Adv. Immunol..

[8] Valecha G.K., Vennepureddy A., Ibrahim U., Safa F., Samra B., Atallah J.P. (2016). Anti–PD-1/PD-L1 antibodies in non-small cell lung cancer: The era of immunotherapy. Expert Rev. Anticancer Ther..

[9] Hunter K.A., Socinski M.A., Villaruz L.C. (2017). PD-L1 Testing in guiding patient selection for PD-1/PD-L1 inhibitor therapy in Lung cancer. Mol. Diagn. Ther..

[10] Spencer K.R., Wang J., Silk A.W., Ganesan S., Kaufman H.L., Mehnert J.M. (2016). Biomarkers for immunotherapy: Current developments and challenges. Am. Soc. Clin. Oncol. Educ. Book.

[11] Bodor J.N., Boumber Y., Borghaei H. (2019). Biomarkers for immune checkpoint inhibition in non-small cell lung cancer (NSCLC). Cancer.

[12] Cyriac G., Gandhi L. (2018). Emerging biomarkers for immune checkpoint inhibition in lung cancer. Semin. Cancer Biol..

[13] Gibney G.T., Weiner L.M., Atkins M.B. (2016). Predictive biomarkers for checkpoint inhibitor-based immunotherapy. Lancet Oncol..

[14] Weber J.S. (2017). Biomarkers for checkpoint inhibition. Am. Soc. Clin. Oncol. Educ. Book.

[15] Fancello L., Gandini S., Pelicci P.G., Mazzarella L. (2019). Tumor mutational burden quantification from targeted gene panels: Major advancements and challenges. J. Immunother. Cancer.

[16] Greillier L., Tomasini P., Barlesi F. (2018). The clinical utility of tumor mutational burden in non-small cell lung cancer. Transl. Lung Cancer Res..

[17] Stenzinger A., Allen J.D., Maas J., Stewart M.D., Merino D.M., Wempe M.M., Dietel M. (2019). Tumor mutational burden standardization initiatives: Recommendations for consistent tumor mutational burden assessment in clinical samples to guide immunotherapy treatment decisions. Genes Chromosom. Cancer.

[18] Ready N., Hellmann M.D., Awad M.M., Otterson G.A., Gutierrez M., Gainor J.F., Borghaei H., Jolivet J., Horn L., Mates M. (2019). First-line nivolumab plus ipilimumab in advanced non–small-cell lung cancer (checkmate 568): Outcomes by programmed death Ligand 1 and tumor mutational burden as biomarkers. J. Clin. Oncol..

[19] Gandara D.R., Paul S.M., Kowanetz M., Schleifman E., Zou W., Li Y., Rittmeyer A., Fehrenbacher L., Otto G., Malboeuf C. (2018). Blood-based tumor mutational burden as a predictor of clinical benefit in non-small-cell lung cancer patients treated with atezolizumab. Nat. Med..

[20] Liu J., Li S., Zhang S., Liu Y., Ma L., Zhu J., Xin Y., Wang Y., Yang C., Cheng Y. (2019). Systemic immune-inflammation index, neutrophil-to-lymphocyte ratio, platelet-to-lymphocyte ratio can predict clinical outcomes in patients with metastatic non-small-cell lung cancer treated with nivolumab. J. Clin. Lab. Anal..

[21] Takeda T., Takeuchi M., Saitoh M., Takeda S. (2018). Neutrophil-to-lymphocyte ratio after four weeks of nivolumab administration as a predictive marker in patients with pretreated non-small-cell lung cancer. Thorac. Cancer.

[22] Thorsson V., Gibbs D., Brown S., Wolf D., Bortone D.S., Yang T.-H.O., Porta-Pardo E., Gao G., Plaisier C.L., Eddy J.A. (2018). The immune landscape of cancer. Immunity.

[23] Bethmann D., Feng Z., A Fox B. (2017). Immunoprofiling as a predictor of patient’s response to cancer therapy—promises and challenges. Curr. Opin. Immunol..

[24] Bremnes R.M., Busund L.-T., Kilvær T.L., Andersen S., Richardsen E., Paulsen E.E., Hald S., Khanehkenari M.R., Cooper W.A., Kao S.C. (2016). The role of tumor-infiltrating lymphocytes in development, progression, and prognosis of non–small cell lung cancer. J. Thorac. Oncol..

[25] Jlert Å.K., Halvorsen A.R., Nebdal D., Lund-Iversen M., Solberg S., Brustugun O.T., Lingjaerde O.C., Helland Å. (2019). The immune microenvironment in non-small cell lung cancer is predictive of prognosis after surgery. Mol. Oncol..

[26] Galon J., Fox B.A., Bifulco C.B., Masucci G., Rau T., Botti G., Marincola F.M., Ciliberto G., Pages F., Ascierto P.A. (2016). Immunoscore and Immunoprofiling in cancer: An update from the melanoma and immunotherapy bridge 2015. J. Transl. Med..

[27] Galon J., Mlecnik B., Bindea G. (2014). Towards the introduction of the ‘Immunoscore’ in the classification of malignant tumours. J. Pathol..

[28] Binnewies M., Roberts E., Kersten K., Chan V., Fearon D.F., Merad M., Coussens L.M., Gabrilovich D.I., Ostrand-Rosenberg S., Hedrick C.C. (2018). Understanding the tumor immune microenvironment (TIME) for effective therapy. Nat. Med..

[29] Ness N., Andersen S., Valkov A., Nordby Y., Donnem T., Al-Saad S., Busund L.-T., Bremnes R.M., Richardsen E. (2014). Infiltration of CD8+ lymphocytes is an independent prognostic factor of biochemical failure-free survival in prostate cancer. Prostate.

[30] Siddiqui S.A., Frigola X., Bonne-Annee S., Mercader M., Kuntz S.M., Krambeck A.E., Sengupta S., Dong H., Cheville J.C., Lohse C.M. (2007). Tumor-infiltrating Foxp3−CD4+CD25+ T cells predict poor survival in renal cell carcinoma. Clin. Cancer Res..

[31] Gnjatic S., Bronte V., Brunet L.R., Butler M.O., Disis M.L., Galon J., Hakansson L.G., Hanks B.A., Karanikas V., Khleif S.N. (2017). Identifying baseline immune-related biomarkers to predict clinical outcome of immunotherapy. J. Immunother. Cancer.

[32] Jensen H.K., Donskov F., Nordsmark M., Marcussen N., Von Der Maase H. (2009). Increased intratumoral FOXP3-positive regulatory immune cells during Interleukin-2 treatment in metastatic renal cell carcinoma. Clin. Cancer Res..

[33] Mella M., Kauppila J.H., Karihtala P., Lehenkari P., Jukkola-Vuorinen A., Soini Y., Auvinen P., Vaarala M.H., Ronkainen H., Kauppila S. (2015). Tumor infiltrating CD8+T lymphocyte count is independent of tumor TLR9 status in treatment naïve triple negative breast cancer and renal cell carcinoma. OncoImmunology.

[34] Anderson A.C., Joller N., Kuchroo V.K. (2016). Lag-3, Tim-3, and TIGIT: Co-inhibitory receptors with specialized functions in immune regulation. Immunity.

[35] Brambilla E., Le Teuff G., Marguet S., Lantuejoul S., Dunant A., Graziano S., Pirker R., Douillard J.-Y., Le Chevalier T., Filipits M. (2016). Prognostic effect of tumor lymphocytic infiltration in resectable non–small-cell lung cancer. J. Clin. Oncol..

[36] Kinoshita T., Kudo-Saito C., Muramatsu R., Fujita T., Saito M., Nagumo H., Sakurai T., Noji S., Takahata E., Yaguchi T. (2017). Determination of poor prognostic immune features of tumour microenvironment in non-smoking patients with lung adenocarcinoma. Eur. J. Cancer.

[37] Lizotte P., Ivanova E.V., Awad M.M., Jones R.E., Keogh L., Liu H., Dries R., Almonte C., Herter-Sprie G.S., Santos A. (2016). Multiparametric profiling of non–small-cell lung cancers reveals distinct immunophenotypes. JCI Insight.

[38] Suzuki K., Kadota K., Sima C.S., Nitadori J., Rusch V.W., Travis W.D. (2013). Clinical impact of immune microenvironment in stage I lung adenocarcinoma: Tumor interleukin-12 receptor β2 (IL-12Rβ2), IL-7R, and stromal FoxP3/CD3 ratio are independent predictors of recurrence. J. Clin. Oncol..

[39] Kawai O., Ishii G., Kubota K., Murata Y., Naito Y., Mizuno T., Aokage K., Saijo N., Nishiwaki Y., Gemma A. (2008). Predominant infiltration of macrophages and CD8+T Cells in cancer nests is a significant predictor of survival in stage IV nonsmall cell lung cancer. Cancer.

[40] Chen D., Wang Y., Zhang X., Ding Q., Wang X., Xue Y., Wang W., Mao Y., Chen C., Chen Y. (2021). Characterization of tumor microenvironment in lung adenocarcinoma identifies immune signatures to predict clinical outcomes and therapeutic responses. Front. Oncol..

[41] Galli F., Aguilera J.V., Palermo B., Markovic S.N., Nisticò P., Signore A. (2020). Relevance of immune cell and tumor microenvironment imaging in the new era of immunotherapy. J. Exp. Clin. Cancer Res..

[42] Seo J.-S., Kim A., Shin J.-Y., Kim Y.T. (2018). Comprehensive analysis of the tumor immune micro-environment in non-small cell lung cancer for efficacy of checkpoint inhibitor. Sci. Rep..

[43] Bonaventura P., Shekarian T., Alcazer V., Valladeau-Guilemond J., Valsesia-Wittmann S., Amigorena S., Caux C., Depil S. (2019). Cold tumors: A therapeutic challenge for immunotherapy. Front. Immunol..

[44] Chi A., He X., Hou L., Nguyen N., Zhu G., Cameron R., Lee J. (2021). Classification of non-small cell lung cancer’s tumor immune micro-environment and strategies to augment its response to immune checkpoint blockade. Cancers.

[45] Gajewski T.F., Corrales L., Williams J., Horton B., Sivan A., Spranger S. (2017). Cancer immunotherapy targets based on understanding the T cell-inflamed versus non-T cell-inflamed tumor microenvironment. Exp. Med. Biol..

[46] Maby P., Bindea G., Mlecnik B., Galon J. (2021). License to kill: Microsatellite instability and immune contexture. OncoImmunology.

[47] Gestermann N., Saugy D., Martignier C., Tillé L., Marraco S.A.F., Zettl M., Tirapu I., Speiser D.E., Verdeil G. (2020). LAG-3 and PD-1+LAG-3 inhibition promote anti-tumor immune responses in human autologous melanoma/T cell co-cultures. OncoImmunology.

[48] Qin S., Xu L., Yi M., Yu S., Wu K., Luo S. (2019). Novel immune checkpoint targets: Moving beyond PD-1 and CTLA-4. Mol. Cancer.

[49] Fan X., Quezada S., Sepulveda M.A., Sharma P., Allison J.P. (2014). Engagement of the ICOS pathway markedly enhances efficacy of CTLA-4 blockade in cancer immunotherapy. J. Exp. Med..

[50] Jayasingam S.D., Citartan M., Thang T.H., Zin A.A.M., Ang K.C., Ch’Ng E.S. (2020). Evaluating the polarization of tumor-associated macrophages into M1 and M2 phenotypes in human cancer tissue: Technicalities and challenges in routine clinical practice. Front. Oncol..

[51] Najafi M., Goradel N.H., Farhood B., Salehi E., Nashtaei M.S., Khanlarkhani N., Khezri Z., Majidpoor J., Abouzaripour M., Habibi M. (2018). Macrophage polarity in cancer: A review. J. Cell. Biochem..

[52] Broz M.L., Binnewies M., Boldajipour B., Nelson A.E., Pollack J.L., Erle D.J. (2014). Dissecting the tumor myeloid compartment reveals rare activating antigen-presenting cells critical for T cell immunity. Cancer Cell.

[53] Gocher A.M., Workman C.J., Vignali D.A.A. (2021). Interferon-γ: Teammate or opponent in the tumour microenvironment?. Nat. Rev. Immunol..

[54] Zhang Y., Guan X.-Y., Jiang P. (2020). Cytokine and chemokine signals of T-cell exclusion in tumors. Front. Immunol..

[55] Weber R., Fleming V., Hu X., Nagibin V., Groth C., Altevogt P., Utikal J., Umansky V. (2018). Myeloid-derived suppressor cells hinder the anti-cancer activity of immune checkpoint inhibitors. Front. Immunol..

[56] Pai S.I., Cesano A., Marincola F.M. (2020). The paradox of cancer immune exclusion: Immune oncology next frontier. Cancer Treat. Res..

[57] Li Y.L., Zhao H., Ren X.B. (2016). Relationship of VEGF/VEGFR with immune and cancer cells: Staggering or forward?. Cancer Biol. Med..

[58] Yang J., Yan J., Liu B. (2018). Targeting VEGF/VEGFR to modulate antitumor immunity. Front. Immunol..

[59] Lloye M.D., Todd W.M. (2014). Therapeutic targeting of cancers with loss of PTEN function. Curr. Drug Targets.

[60] Peng W., Chen J.Q., Liu C., Malu S., Creasy C., Tetzlaff M.T., Xu C., McKenzie J.A., Zhang C., Liang X. (2015). Loss of PTEN promotes resistance to T cell–mediated immunotherapy. Cancer Discov..

[61] Gil-Julio H., Perea F., Rodriguez-Nicolas A., Cozar J.M., González-Ramirez A., Concha A., Garrido F., Aptsiauri N., Ruiz-Cabello F. (2021). Tumor escape phenotype in bladder cancer is associated with loss of HLA class I expression, T-cell exclusion and stromal changes. Int. J. Mol. Sci..

[62] Riaz N., Havel J., Makarov V., Desrichard A., Urba W.J., Sims J.S., Hodi F.S., Martín-Algarra S., Mandal R., Sharfman W.H. (2017). Tumor and microenvironment evolution during immunotherapy with nivolumab. Cell.

[63] Kümpers C., Jokic M., Haase O., Offermann A., Vogel W., Grätz V., Langan E.A., Perner S., Terheyden P. (2019). Immune cell infiltration of the primary tumor, not PD-L1 status, is associated with improved response to checkpoint inhibition in metastatic melanoma. Front. Med..

[64] Lam V.K., Zhang J. (2019). Blood-based tumor mutation burden: Continued progress toward personalizing immunotherapy in non-small cell lung cancer. J. Thorac. Dis..

[65] van Campenhout C., Meléndez B., Remmelink M., Salmon I., D’Haene N. (2019). Blood tumor mutational burden: Are we ready for clinical implementation?. J. Thorac. Dis..

[66] Hellmann M.D., Nathanson T., Rizvi H., Creelan B.C., Sanchez-Vega F., Ahuja A., Ni A., Novik J.B., Mangarin L.M., Abu-Akeel M. (2018). Genomic features of response to combination immunotherapy in patients with advanced non-small-cell lung cancer. Cancer Cell.

[67] Hwang S., Kwon A.-Y., Jeong J.-Y., Kim S., Kang H., Park J., Kim J.-H., Han O.J., Lim S.M., An H.J. (2020). Immune gene signatures for predicting durable clinical benefit of anti-PD-1 immunotherapy in patients with non-small cell lung cancer. Sci. Rep..

[68] Althammer S., Tan T.H., Spitzmüller A., Rognoni L., Wiestler T., Herz T., Widmaier M., Rebelatto M.C., Kaplon H., Damotte D. (2019). Automated image analysis of NSCLC biopsies to predict response to anti-PD-L1 therapy. J. Immunother. Cancer.

[69] Bruni D., Angell H.K., Galon J. (2020). The immune contexture and immunoscore in cancer prognosis and therapeutic efficacy. Nat. Rev. Cancer.

